# Plant–soil interactions in the native range of two congeneric species with contrasting invasive success

**DOI:** 10.1007/s00442-023-05329-6

**Published:** 2023-02-06

**Authors:** Anna Florianová, Věra Hanzelková, Lucie Drtinová, Hana Pánková, Tomáš Cajthaml, Zuzana Münzbergová

**Affiliations:** 1 Department of Botany, Faculty of Science, Charles University in Prague, Benátská 2, 128 01 Prague, Czech Republic; 2 Institute of Botany of the Czech Academy of Sciences, Zámek 1, 252 43 Průhonice, Czech Republic; 3 Institute of Microbiology of the Czech Academy of Sciences, Vídeňská, 1083, 142 20 Prague, Czech Republic; 4 Institute for Environmental Studies, Faculty of Science, Charles University in Prague, Benátská 2, 128 01 Prague, Czech Republic

**Keywords:** Arbuscular mycorrhizal fungi (AMF), Enemy release hypothesis, Mycorrhizal inoculation potential (MIP), Phospholipid/neutral fatty acid analysis (PLFA/NLFA), Plant invasiveness, Structural equation modelling

## Abstract

The aim of this study was to compare plant–soil interactions in the native range of two congeneric European species differing in their invasive success in the world: a globally invasive *Cirsium vulgare* and non-invasive *C. oleraceum*. We assessed changes in soil nutrients and soil biota following soil conditioning by each species and compared performance of plants grown in self-conditioned and unconditioned soil, from which all, some or no biota was excluded. The invasive species depleted more nutrients than the non-invasive species and coped better with altered nutrient levels. The invasive species had higher seedling establishment which benefited from the presence of unconditioned biota transferred by soil filtrate. Biomass of both species increased in soil with self-conditioned soil filtrate and decreased in soil with self-conditioned whole-soil inoculum compared to unconditioned filtrate and inoculum. However, the increase was smaller and the decrease greater for the invasive species. The invasive species allocated less biomass to roots when associated with harmful biota, reducing negative effects of the biota on its performance. The results show that in the native range the invasive species is more limited by self-conditioned pathogens and benefits more from unconditioned mutualists and thus may benefit more from loss of effectively specialized soil biota in a secondary range. Our study highlights the utility of detailed plant-soil feedback research in species native range for understanding factors regulating species performance in their native range and pinpointing the types of biota involved in their regulation.

**Supplementary Information:**

The online version contains supplementary material available at 10.1007/s00442-023-05329-6.

## Introduction

Understanding the success of invasive species and why some alien plants become invasive while others fail is a fundamental goal in the field of invasion ecology. Despite a high number of hypotheses explaining the success of invasive species, such as the enemy release hypothesis (Enders et al. 2020; Keane and Crawley 2002), we are far from fully comprehending what drives successful invasion. A promising approach to understanding the mechanisms that allow for invasion is to understand the factors that regulate species performance in the native range. For example, invasive species might be those that take advantage of resource-rich environments by rapidly taking-up and depleting available resources (Gioria and Osborne 2014; Mathakutha et al. 2019), but in their native range become limited by specialized (specialist or ‘effectively specialized’, see Semchenko et al. 2022) enemies, e.g. various soil pathogens, once they become abundant (Zuppinger-Dingley et al. 2011). In their secondary range, these species might be less limited by the specialized soil-borne enemies leading to possibly less negative conspecific plant-soil feedback, giving them advantage over other species (Aldorfova et al. 2020; Klironomos 2002; Kulmatiski et al. 2008; Suding et al. 2013).

Plant-soil feedback (PSF) occurs when plants alter soil properties that subsequently influence the performance of plants (Bever et al. 1997), and is typically quantified as a log-response ratio of plant performance in self-conditioned soil to performance in control (unconditioned or heterospecific-conditioned) soil (Brinkman et al. 2010). PSF has been suggested to play a role in plant invasions. In their secondary range, invasive species often experience more positive (or less negative) PSF compared to native species (Chiuffo et al. 2015; Engelkes et al. 2008; van der Putten et al. 2007), and PSF of many invasive species has also been shown to be more positive (or less negative) in their secondary than in their native range (Callaway et al. 2011; Reinhart and Callaway 2004; Reinhart et al. 2003).

To assess the importance of PSF for the success of invasive species, we need comparisons of successful invaders with less-successful species that only naturalize in the secondary range. Such studies are scarce. Aldorfova et al. (2020) showed that in the secondary range, invasive species experience more positive (or less negative) PSF than non-invasive alien species. Montesinos and Callaway (2020) and McGinn et al. (2018) compared PSF of invasive and non-invasive congeners in their native and secondary ranges, the first showing a greater release from negative PSF in the secondary range for the invasive species, the other reporting no difference among species with different invasive success. Particularly little is known about differences in PSF of invasive species and their non-invading relatives in their native ranges. Possibly, patterns by which species interact with soil in their native range determine the degree to which they benefit from pathogen release when introduced to the secondary range and thus determine their chances of becoming invasive. In support of this hypothesis, Zuppinger-Dingley et al. (2011) showed on a set of 16 grassland species that species invasive in some region of the world show more negative PSF in their native range than their non-invading relatives. More studies contrasting PSF of invasive and non-invasive species in their native range are needed to assess the importance of plant–soil interactions in determining invasive success of alien plant species.

Plant–soil feedback includes species-specific modifications of both abiotic and biotic soil properties. Many different taxa of soil biota contribute to the biotic component of PSF, including bacteria, arbuscular mycorrhizal fungi (AMF), non-mycorrhizal fungi, protozoans, and nematodes. These groups can all have positive, negative or net neutral effects on the plants, and we are still far from understanding to what extent they contribute to PSF. To assess how specific soil biota affects plant performance, the ideal approach would be to isolate the biota, prepare pure cultures, and inoculate plants with them, which is, however, extremely time-consuming (Dawson and Schrama 2016). An easier, yet still valuable approach is to take advantage of the fact that soil biota largely varies in size and inoculate the soil with whole or partial soil biota obtained by filtering soil solutions. By doing so, one can for example, at least partly, separate the effects of soil microbiota (non-mycorrhizal fungi and bacteria) from the effects of larger-sized soil biota, including AMF and nematodes (van de Voorde et al. 2012; Wang et al. 2019a, 2019b). Even though some AMF spores and nematode eggs likely pass even through the smallest sieve mesh (Wang et al. 2019a) and the filtration approach thus does not create perfectly separated groups of functionally similar taxa, the method has still been recognized as a useful tool for creating a richness gradient in soil communities (Wagg et al. 2014, 2019). Comparing effects of different soil fractions on plant growth can therefore bring additional insight into the drivers of PSF compared to studies only using whole-soil inoculum assays (van de Voorde et al. 2012).

Most studies address PSF solely in terms of aboveground biomass of adult plants as it is the easiest measure of plant performance (Kardol et al. 2013). However, it has been hypothesized (Kardol et al. 2013) and shown (Aldorfova et al. 2020; Dudenhoffer et al. 2018; Florianova and Munzbergova 2018) that PSF effects on germination, survival or establishment of seedlings can differ in intensity and even in direction from PSF effects on biomass of adult plants. In addition, PSF alters the size of the root system and allocation to root biomass (Aldorfova and Munzbergova 2019; Bergmann et al. 2016; Cortois et al. 2016; Hendriks et al. 2015; Wilschut and van Kleunen 2021), which further affect plant performance and ability to deal with environmental stress. Including multiple measures of plant performance in PSF studies is thus needed to better understand the overall effects of soil biota on plant fitness.

Here, we studied *Cirsium vulgare* (Asteraceae), a species that is native to Europe, but has successfully naturalized on every continent except Antarctica, and is considered highly invasive in some areas, particularly in North America (Julien and Griffiths 1998; Tenhumberg et al. 2008). We compared plant–soil interactions of *C. vulgare* (hereafter the invasive species) in its native range in Europe with plant–soil interactions of its native congener, *C. oleraceum* that is not known to be invasive anywhere in the world (hereafter the non-invasive species). Specifically, we used structural equation modeling to understand how the abiotic and biotic pathways in plant––soil interactions influence plant performance (measured as seedling establishment, aboveground biomass, and root–shoot ratio). This required quantifying (i) how the species condition the soil, both in respect to changes in soil nutrient levels and composition of soil biota, (ii) how plant performance responds to soil conditioning (using multiple soil treatments – sterilized soil, self-conditioned or unconditioned soil filtrate or whole-soil inoculum, and whole soil), and (iii) which groups of soil biota (e.g., bacteria, non-mycorrhizal fungi, AMF) are driving these changes in plant performance. We expected that the invasive species will act as follows: (i) deplete more soil nutrients and accumulate more soil biota during soil conditioning than the non-invasive species, (ii) be more negatively affected by growth with self-conditioned biota compared to unconditioned biota than the non-invasive species, allowing it to benefit more from release from the biota when introduced to a secondary range; and (iii) be more negatively affected by soil bacteria and non-mycorrhizal fungi, but will not differ in response to AMF from the non-invasive species.

## Material and methods

### Studied species and seed collection

We selected a pair of sympatric congeneric species, *Cirsium vulgare* (native to Europe, invasive elsewhere, hereafter the invasive species) and *Cirsium oleraceum* (native to Europe, not known to be invasive anywhere, hereafter the non-invasive species)*,* Asteraceae, Carduoideae, for this study. Both species are native to Europe and act as ruderal species, i.e. often colonize disturbed lands. Both species are common in the Czech Republic where the study was performed, with *C. oleraceum* being more frequent and abundant than *C. vulgare* (occurrence frequency in vegetation plots 4.1% vs 1%, mean percentage cover 7.8% vs 2.3%, and maximum percentage cover 88% vs 38%, Wild et al. 2019). However, globally, and especially in North America, *C. vulgare* is reported to be a noxious weed and highly invasive species (Julien and Griffiths 1998; Sieg et al. 2003; Tenhumberg et al. 2008), while *C. oleraceum* has never been reported as an invader outside the native range.

We collected seeds of both species in the field in Central Bohemia, Czech Republic in 2017, from three different populations at least 3 km apart. For each population, we collected mature seeds from at least 10 individuals that were at least 2 m apart from each other. We mixed seeds from all individuals and populations and did not further distinguish mother plants in the experiment. All collected seeds were surface sterilized with 10% dilution of SAVO Originál (a disinfectant based on 4.7% sodium hypochlorite) prior to the experiment to reduce the chance of soil contamination via seed surface fungi, as commonly done in experiments (e.g., Gallery et al. 2007; Oono et al. 2020).

### Experimental design

**Fig. 1:**
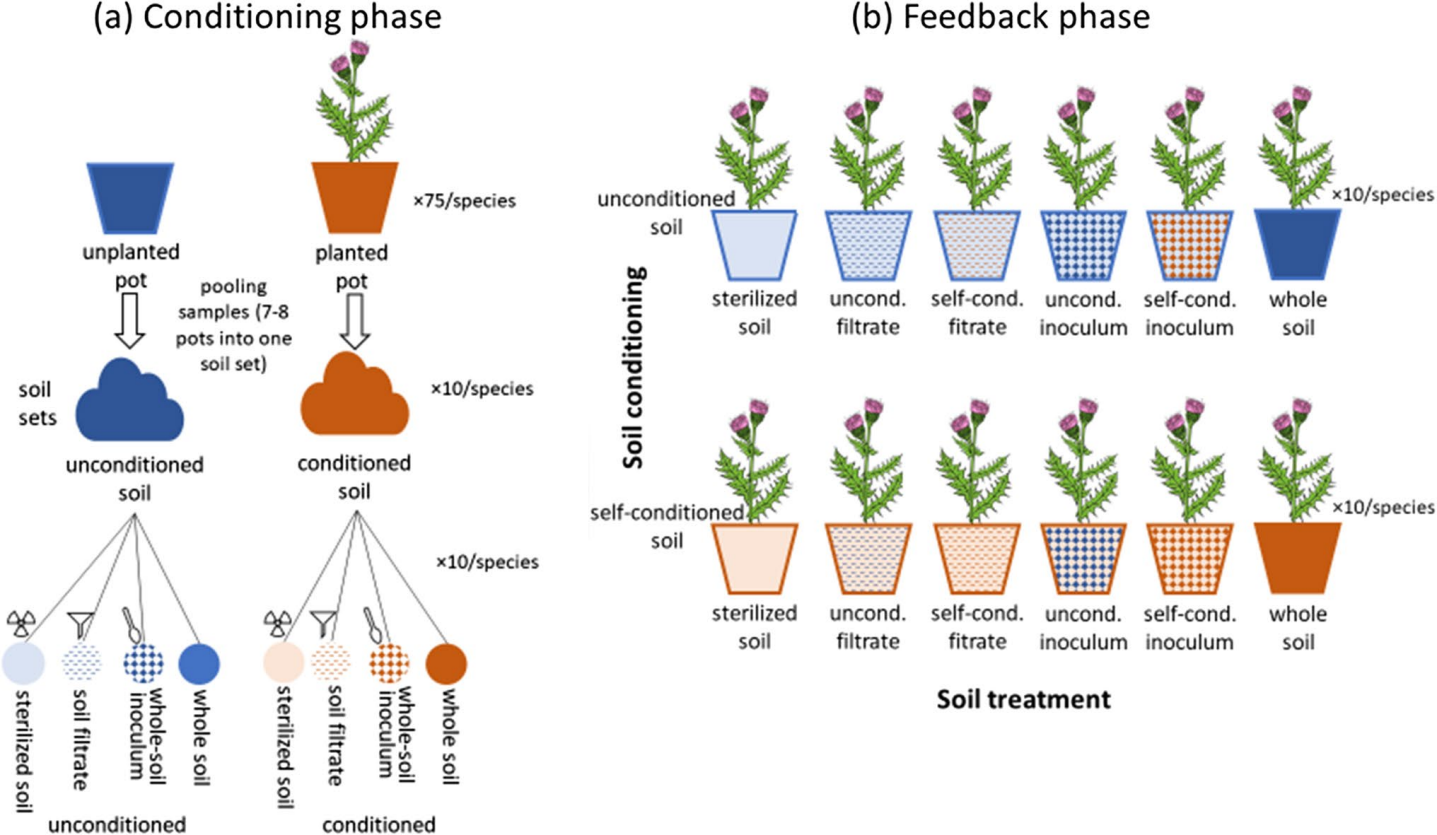
A schematic diagram of the experimental design. **a** During the conditioning phase, we grew each of the two study species in 75 pots in mixture of top-soil and sand to create conditioned soil. The same soil mixture was left in unplanted pots under the same conditions to serve as control (unconditioned) soil. Soil from 7–8 pots was pooled to create 10 sets of unconditioned and 10 sets of self-conditioned soil for each species. Each soil set was used to obtain sterilized soil (gamma irradiation, 25 kGy), soil filtrate (filtering soil suspension through two filter papers with pore size of 11 μm), whole-soil inoculum (sterilized soil and untreated soil in 9:1 ratio), and untreated whole soil. **b** During the feedback phase, we grew each species in six treatments of unconditioned and six treatments of self-conditioned soil (2 levels of soil conditioning). These treatments included sterilized soil, sterilized soil inoculated with soil filtrate from unconditioned or self-conditioned soil, sterilized soil inoculated with whole-soil inoculum from unconditioned or self-conditioned soil, and non-sterilized whole soil

Following a commonly used methodology (Bever et al. 1997; Kulmatiski et al. 2008), we grew the plants in a two-phase experiment. In the first (conditioning) phase, soil conditioned by each species was prepared. We assessed soil abiotic and biotic characteristics after the conditioning phase to compare the effect of the two species on soil. In the second (feedback) phase, we assessed the effect of self-conditioned and unconditioned control soil and soil biota on plant growth. Contrary to most PSF experiments that only compare plant performance in self-conditioned and control (unconditioned or heterospecific-conditioned) soil (total PSF) or in sterilized soil into which either self-conditioned or control whole-soil inoculum is added (biotic PSF), we used 12 different soil treatments in the feedback phase (Fig. 1). These treatments included self-conditioned and unconditioned whole soil, and self-conditioned and unconditioned sterilized soil into which no biota, partial soil biota (soil filtrate) or all biota (whole-soil inoculum) from either self-conditioned or unconditioned soil were added. Such experimental design allowed us to assess the effects of changes in both abiotic and biotic soil properties and to distinguish between the effect of presence of soil biota transferred by individual soil fractions (soil filtrate or whole-soil inoculum vs sterilized soil, ‘total PSF’ sensu Semchenko et al. 2022) and of conditioning of the soil biota (self-conditioned vs unconditioned filtrate or inoculum, ‘specific PSF’ sensu Semchenko et al. 2022). Because multiple comparisons can be drawn using our data, we decided not to quantify PSF as a log-response ratio as commonly done in PSF studies (Brinkman et al. 2010), but decided to present raw data on plant performance in individual treatments instead. For more details on what information is provided by each comparison, see Table 1.

**Table 1:**
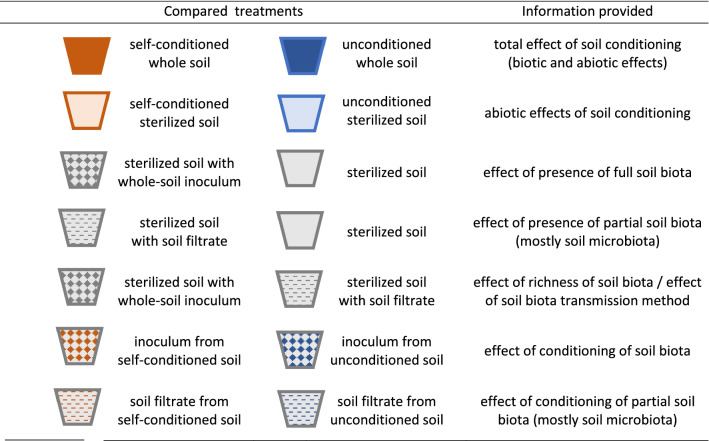
Information provided by different treatment comparisons

We used unplanted (unconditioned) soil as control, similarly to a range of previous studies (e.g., Aldorfova et al. 2022, 2020; Kardol et al. 2007; Kulmatiski et al. 2011; Perkins and Nowak 2013; Wang et al. 2013). This approach allowed us to evaluate the effect of specific changes in both biotic and abiotic soil characteristics, not confounded by specific effects of selected control species when using heterospecific-conditioned soil as control (Brinkman et al. 2010; Kulmatiski and Kardol 2008). Using unplanted soil as control is especially suitable for studying PSF of our study species as they often colonize disturbed habitats and thus frequently encounter bare soil. However, it is important to note that the unconditioned soil likely had lower levels of soil biota due to some plant-obligate biota dying in an unplanted soil and that using a different type of control could provide different results. When interpreting the results or comparing them with other studies, we thus need to keep in mind the type of control in each specific study.

### Conditioning phase

The aim of the conditioning phase was to prepare the soil, conditioned by the species, for the upcoming feedback phase. The conditioning phase was carried out between April 2018 and July 2018 in the experimental garden of the Institute of Botany of the Czech Academy of Sciences (49°59′38.972′′N, 14°33′57.637′′E), 320 m above sea level, temperate climate zone, where the mean annual temperature is 8.6 °C and the mean annual precipitation is 610 mm.

To set up the conditioning phase, we used a mixture of topsoil (purchased from JENA company) and sand (AGRO Jesenice) in 1:1 ratio. The topsoil originated in Central Bohemia, Czech Republic (i.e. the region of seed collection), and was previously grown with a mixture of grassland plant species (information on the exact history of the soil, such as which plant species were grown in it and for how long, has not been provided by the company). For chemical characteristics of the soil mixture see Table S1. For each species, we used 150 1-L pots (10 × 10 × 10 cm) in the conditioning phase. Half of the pots were sown with 10 seeds of one of the species in April 2018, the other half of the pots remained unsown and served as controls (unconditioned soil). Even though the control pots remained unplanted during the conditioning phase, the soil mixture contained 50% of the live topsoil in which a mixture of grassland plant species was previously grown, and it thus contained species non-specific (unconditioned) soil biota. Each pot with conditioned soil was randomly assigned its unplanted control pot. The pairs of pots were always kept next to each other throughout the experiment so that they were exposed to as similar conditions as possible. To prevent cross-contamination, the pots were filled to 1.5 cm below the top, each pot was placed in an individual sterile plastic saucer, and the pots were placed ~ 10 cm apart. Both pots with and without plants were kept under the same conditions, regularly watered with tap water, and weeded on a weekly basis to avoid any effects of other species on the soil.

After the seeds germinated and the seedlings established, pots were thinned to one largest seedling. The soil was conditioned for 12 weeks, similar to a range of previous studies (e.g., Chiuffo et al. 2015; Florianova and Munzbergova 2018; Meijer et al. 2011; van de Voorde et al. 2011; van Grunsven et al. 2007; van Grunsven et al. 2010). Twelve weeks should be a sufficient time for the soil to get properly conditioned, particularly for small pots we used in our study, as all of the soil was thoroughly colonized by the roots by the end of the 12th week. A previous study showed that PSF is getting more negative with increasing duration of the conditioning phase only up to 6 weeks of conditioning (Lepinay et al. 2018). In addition, it has been shown that the initial changes in soil microbial composition very well reflect the long-term changes (Hannula et al. 2021). After the 12 weeks, in July 2018, all plants were harvested, divided into aboveground and belowground parts (all larger roots were carefully taken out from the soil by hand), dried to a constant weight, and weighed. Cross-contamination of soils from different pots during harvest was avoided by cleansing all used material in 70% ethanol in between working steps. Plants in the conditioning phase showed very little variation in biomass among pots, and the data on their biomass was thus not used in any further analyses.

After the harvest, the 75 pots with each species as well as their paired unplanted control pots (75 pots) were randomly divided into 10 groups of 7–8 pots and soil from these pots was mixed, creating a soil set (a pooled soil sample). For each species we thus had 10 sets of self-conditioned soil and 10 paired sets of unconditioned soil which served as replicates and were analyzed for soil chemistry and soil biota. Pooling the soil samples reduced the pot-to-pot variation in soil biota, but ensured sufficient amount of soil available for the analyses and for all treatments in the feedback phase while reducing cost of the analyses and preserving some level of true independent replication (*n* = 10 per treatment), in line with recommendations (Peacher and Meiners 2020; Rinella and Reinhart 2019). From each soil set, one sixth of the soil was collected for analysis of soil chemistry and soil biota, one-third was kept untreated to serve as source of self-conditioned or unconditioned biota for soil inoculation in the feedback phase, and the rest was sterilized by gamma irradiation (sterilization dose 25 kGy, performed by Bioster a.s. in Veverská Bítýška, Czech Republic) and used as background soil in the feedback phase. Before use in the feedback phase, the sterilized soil was placed in pots and repeatedly watered with controlled amount of distilled water to leach nutrients released by the sterilization procedure.

### Feedback phase

The feedback phase was set up in September 2018 in a greenhouse of the Institute of Botany of the Czech Academy of Sciences. The greenhouse was heated to 18 °C and light regime was set to reflect natural daylight conditions during the main vegetation season. Specifically, because the feedback phase started late in the vegetation season and partly ran through winter, we used actual daylight extended by two hours every day.

In the feedback phase, we grew each species in six treatments of unconditioned soil and in six treatments of self-conditioned soil. These treatments included sterilized soil, sterilized soil inoculated with soil filtrate (i.e. partial soil biota, mostly microbiota) from unconditioned or self-conditioned soil, sterilized soil inoculated with whole-soil inoculum (i.e. full soil biota) from unconditioned or self-conditioned soil, and non-sterilized whole soil (Fig. 1). Inoculum and filtrate always originated from the same soil set as the sterilized background soil or from the paired soil set with different soil conditioning. Using these treatments allowed us to disentangle the effects of abiotic changes in soil due to soil conditioning and the effects of presence and conditioning of soil biota on plant performance (Table 1).

To set up the feedback phase, we used 10 1-L pots (10 × 10 × 10 cm) per species, soil conditioning and treatment, resulting in 120 pots per species and 240 pots in total. The number of pots in the feedback phase was smaller than in the conditioning phase because part of the soil from the conditioning phase was used for analyses of soil chemistry, soil biota and infection potential of AMF. The bottoms of the pots were covered with expanded clay pebbles (keramzit), sterilized in autoclave, up to the height of 2 cm to compensate for soil lost during the harvest of the conditioning phase, and the rest of the pots was filled with 500 ml of soil mixed depending on the treatment. For the whole soil treatments, we used untreated soil from the conditioning phase of the experiment. For whole-soil inoculum treatments, we mixed sterilized soil and untreated soil from the conditioning phase in a 9:1 ratio. For the treatments with soil filtrate, we filled the pots with sterilized soil and we watered them with the soil filtrate. The filtrate was created by mixing 50 ml of untreated soil in 500 ml of distilled water, homogenizing the mixture, and filtering it through two filter papers with pore size of 11 μm. The soil filtrate should therefore contain mostly soil microbiota, i.e. bacteria and non-mycorrhizal fungi, and it should not contain microarthropods, nematodes, or AMF (van de Voorde et al. 2012), even though some nematode eggs or AMF spores could have passed through the filter papers as well (Wang et al. 2019a). For sterilized treatments, we filled the pots with sterilized soil and watered them with autoclaved soil filtrate.

Each pot was sown with 9 seeds of one of the species (seeds were sown into self-conditioned or unconditioned soil only, not into heterospecific-conditioned soil). The pots were kept in the greenhouse, regularly watered, and weeded when needed. All pots originating from one pair of soil sets were kept in the same block within the greenhouse. Number of emerged seedlings was recorded on a weekly basis. Three weeks after the first seedlings emerged in all pots, all seedlings but the largest one were removed from each pot to avoid competition. All seedlings emerging afterwards were recorded and removed as well. Twelve weeks after germination, the plants were harvested, divided into above- and below-ground biomass and weighed. All plants of both species were harvested at the same time.

### Soil characteristics

Soil characteristics (i.e. soil abiotic characteristics, soil microbial community, and infection potential of AMF, see below) were analyzed after the conditioning phase for three types of soil: soil conditioned by the invasive species, soil conditioned by the non-invasive species, and the unconditioned soil. For each of the soil conditioning types, samples from six out of the ten soil sets were randomly selected for the analyses. In addition, the analyses were performed also on soil collected before the conditioning phase (Table S1).

For abiotic soil characteristics, we measured actual pH (pH measured in deionized H_2_O) and exchangeable pH (cation exchange capacity), total C, N, P and available P, Ca, Mg, and K. For biotic characteristics, we determined soil microbial community composition using phospholipid and neutral fatty acid analysis (PLFA and NLFA) and we assessed the infection potential of AMF by the following commonly used procedures: the most probable number (MPN, Adelman and Morton 1986; Wilson and Trinick 1983) and mean infection percentage (MIP, Giovannetti and Mosse 1980; Moorman and Reeves 1979).

### Abiotic soil characteristics

Actual and exchangeable pH was measured using deionized water and 0.1 M solution of KCl as extracting solutions, respectively (ISO 10390: Soil quality – Determination of pH. International Organization for Standardization, ISO 2000). Total C and N contents were determined by methods of Ehrenberger and Gorbach (1973) using CHN catalyst (Carlo Erba NC 2500), total P was measured according to the method of Olsen and Sommers (1982). Available P was measured in filtrate of 5 g of air dried soil with 50 ml of 0.5 M K_2_SO_4_ solution by flow injection analysis with spectrophotometric detection using the instrument QuikChem FIA + 8000 Series (Ammerman 2001; Egan 2001). Concentrations of available Ca^2+^ and K^+^ were measured using atomic emission spectrometry method and available Mg^2+^ using atomic absorption spectrometry according to methods of Moore and Chapman (1986) and Dědina (1987), with solution of 1 M ammonium acetate as the extractant. All analyses were performed by the Analytical Laboratory of Institute of Botany of the Czech Academy of Sciences in Průhonice.

### Soil microbial community

Soil microbial community composition was assessed using PLFA analysis performed by the Laboratory of Environmental Biotechnology, Institute of Microbiology of the Czech Academy of Sciences, following the methodology described in Garcia-Sanchez et al. (2019). The PLFA were extracted from 1 g of freeze-dried soil samples with a mixture of chloroform–methanol-phosphate buffer (1:2:0.8, v/v/v), as previously described by Bligh and Dyer (1959). The lipids were fractionated into neutral lipids (NLFA), glycolipids and polar lipids (PLFAs) using an extraction cartridge (LiChrolut Si-60, Merck, White-house Station, USA), and NLFA and PLFA were subjected to mild alkaline methanolysis as described in Snajdr et al. (2008). The free methyl esters of NLFA and PLFAs were analyzed by gas chromatography-mass spectrometry (450-GC, 240-MS ion trap detector, Varian, Walnut Creek, CA) following the same procedure described by Sampedro et al. (2009).

The soil microbial community composition was characterized using the following PLFAs: fungal biomass was estimated on the basis of 18:2w6,9 content (Snajdr et al. 2008), bacterial biomass was quantified as the sum of i14:0, i15:0, a15:0, 16:1w5, 16:1w7; 16:1w9, 10Me-16:0, i16:0, i17:0, a17:0, cy17:0, 17:0, 10Me-17:0, 18:1w7, 10Me-18:0, and cy19:0. Actinobacterial biomass was determined as the sum of 10Me-16:0, 10Me-17:0, and 10-Me18:0, Gram-positive bacteria (G +) as sum of i14:0, i15:0, a15:0, i16:0, i17:0, and a17:0, and Gram-negative bacteria (G-) as the sum of 16:1w7, 16:1w9, 18:1w7, cy17:0, and cy19:0. The NLFA 16:1w5 was assigned as a marker for the quantification of AMF and total PLFA concentration was used to estimate the total viable microbial biomass (Olsson et al. 2003). Last, we calculated microbial ratios F:B (fungi: bacteria), G + :G- (Gram-positive bacteria: Gram-negative bacteria), and F:AMF (fungi: AMF).

### Infection potential of AMF

Infection potential of AMF was assessed using MIP and MPN methods. In the MIP assay, the colonization intensity of AMF is measured after a certain period of bait plant cultivation and the index of root colonization is the percentage of the number of 1-cm root segments showing detectable AMF colonization (Moorman and Reeves 1979). In MPN method, the test plants are grown in serial dilutions of the inoculum and the propagule density of the original material is statistically calculated from MPN scores (Feldmann and Idczak 1992).

To assess MPN and MIP, we evaluated mycorrhizal colonization of maize roots (standardly used for assessing MPN and MIP as its roots are strongly colonized by AMF (Moorman and Reeves 1979) that was grown in each of the studied types of soil in 1:0, 1:10, 1:100, 1:1000, 1:10,000 dilutions with soil sterilized in autoclave, in five replicates per dilution. Maize seeds (*Zea mays* convar. *saccharata*, var. Ashworth) were purchased from a commercial supplier (ReinSaat KG company, St. Leonhard am Hornerwald, Austria), they were germinated in Petri dishes in sterile conditions, replanted into 100 ml plastic containers (4 × 14 cm), and left growing in a greenhouse. After six weeks, the plants were harvested, fine roots from the middle part of the root system were collected, placed in 10% KOH for three months to bleach, and stained (left for 12 h in 2% lactic acid, 12 h in 0.05% trypan blue in lactoglycerol, rinsed in water, and soaked into lactoglycerol prepared from glycerol, 80% lactic acid and distilled water in 3:2:5 ratio).

The stained roots from 1:10, 1:100, 1:1000, and 1:10,000 dilutions were observed using a binocular magnifier and presence of AMF propagules was recorded. MPN/ml was calculated using a program MPN Calculator, Build 23 using information on types of dilutions, number of replicates per dilution and number of replicates per dilution in which AMF propagules were recorded. To assess MIP, only the 1:10 dilution was used. Stained roots were placed into a Petri dish with a 1 × 1 cm grid and presence of AMF propagules at 200 intersections of roots with the grid was recorded using a binocular magnifier. An average value from the five replicates was calculated both for MPN and MIP, resulting in one MPN and one MIP value per soil sample and six replicates per soil conditioning type.

### Statistical analyses

Differences in soil abiotic and biotic characteristics between soil conditioned by the invasive and the non-invasive species were studied using linear direct gradient analysis (Redundancy Analysis, RDA) and Monte-Carlo permutation tests (Ter Braak and Šmilauer 2012) with 499 permutations. Dependent variables used in this analysis were all the studied soil characteristics except for actual pH, K content, total microbial and bacterial biomass, which were excluded due to high correlations with other variables (Table S2). The variables were standardized prior to the analysis. The independent variable (fixed factor) was the conditioning species. Contrary to our original plan, we did not consider plant biomass from the conditioning phase as a covariate in the analysis due to very little variation in the data. We repeated the analysis with all three soil conditioning types including the unconditioned soil and we present the results in the appendix (Fig. S1). The analyses were performed in Canoco 5 (Ter Braak and Šmilauer 2012). As a supplementary analysis, we also performed ANOVA using R 3.6.1 (R Core Team 2019), always with one of the studied soil characteristics as a dependent variable, and we tested the differences between pairs of group means using Tukey post hoc tests (Fig. S2).

Differences in plant performance between individual treatments and soil conditioning types in the feedback phase were tested using a linear (square root transformed biomass and root-shoot ratio) or generalized linear (seedling establishment as the number of established seedlings out of the number of seeds sown, with binomial error distribution) mixed effect models in the R-package ‘lmerTest’ (Kuznetsova et al. 2017). Soil set was considered a random effect and species, soil conditioning (unconditioned vs self-conditioned soil), soil treatment (sterilized soil, filtrate from unconditioned soil, filtrate from self-conditioned soil, inoculum from unconditioned soil, inoculum from self-conditioned soil, whole soil), and their interactions as fixed effects. To estimate p-values, we used F-tests comparing two models with and without a tested term, using a ‘drop1’ function in the ‘lmerTest’ package (Kuznetsova et al. 2017). To assess differences between pairs of group means, we used Tukey post-hoc tests adapted to mixed effect models using ‘glht’ function in ‘multcomp’ R-package (Hothorn et al. 2008).

Afterwards, we repeated the analysis using the type of soil biota (partial soil biota from soil filtrate vs full soil biota from whole-soil inoculum) and conditioning of soil biota (from unconditioned vs self-conditioned soil) as explanatory variables instead of the treatment variable. We performed this analysis on a subset of data excluding the sterilized and the whole soil treatment, since in these treatments it is not possible to distinguish between the effects of type and conditioning of soil biota. We obtained very similar results when including only the subset of treatments in the analyses and we therefore only present these results in the Results section. Results of the analyses including all the treatments and not differentiating between type and conditioning of soil biota can be found in the appendix (Table S3). The two treatments which are excluded from the main analyses are, however, visualized in some of the graphs and compared using multiple comparisons with the rest.

Last, we used structural equation modeling (performed in the ‘lavaan’ R-package, Rosseel 2012) to assess how individual components of soil, i.e. amount of soil nutrients, bacterial, fungal and AMF biomass, affect biomass of the two species. For the analysis, we only used data on plant biomass from the whole soil treatment as detailed soil analyses are only available for this treatment. A separate model was created for each species. The assumed relationships were as follows: (i) plant performance is affected by the amount of soil nutrients and by bacterial, fungal and AMF biomass, (ii) bacterial, fungal and AMF biomass are affected by the amount of soil nutrients, and (iii) bacterial, fungal and AMF biomass are correlated.

## Results

### Effect of conditioning species on soil characteristics

**Fig. 2:**
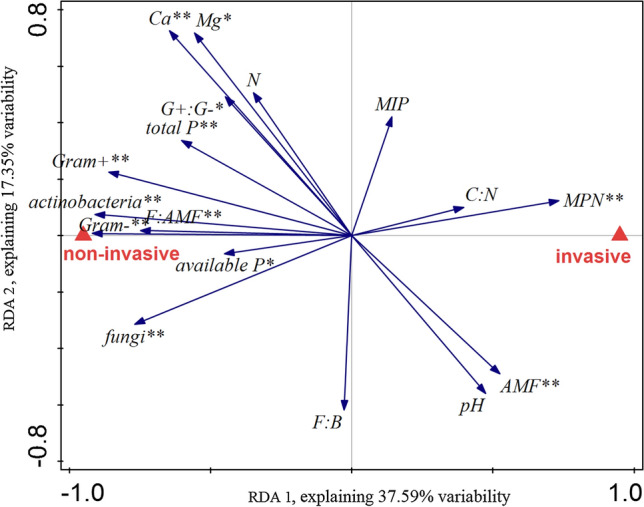
Differences in soil abiotic and biotic characteristics in soils conditioned by the invasive and the non-invasive sympatric congeneric species. Results displayed are an ordination plot based on RDA tested using a Monte-Carlo test with 499 permutations. Pseudo-F = 6.0, *p* = 0.004, the first two axes explained 37.59% and 17.35% variability in the data, respectively. Data are centered and standardized across soil characteristics. **indicates characteristics that are significantly (*p* < 0.05) different between the two soil types in individual tests, *indicates marginally significant (*p* < 0.1) differences. Actinobacteria, Gram + , Gram-, fungi, and AMF represent biomass of the groups obtained by PLFA/NLFA analyses. G + :G-, F:AMF, and F:B represent ratios of Gram positive (G +) and Gram negative (G-) bacteria, fungi (F), AMF and bacteria (B)

Soil characteristics after the conditioning phase significantly differed between soils conditioned by the invasive and by the non-invasive species (Pseudo-F = 6.0, *p* = 0.004, 37.59% of explained variation, Fig. 2). Values of MPN and AMF biomass were higher in soils conditioned by the invasive species (Fig. 2, Fig. S2), and in both cases the values were much higher than in the unconditioned soil (Fig. S1, S2). Nutrient levels and both bacterial and fungal biomass were higher in soils conditioned by the non-invasive species (Fig. 2, Fig. S2).

### Effect of soil conditioning and treatments on plant performance in the feedback phase

In the feedback phase, the invasive species had nearly four times higher overall seedling establishment and two times lower root-shoot ratio than the non-invasive species (Table 2, Fig. S3). The two species did not differ in overall biomass production (across all treatments, Table 2).

**Table 2:** Results of generalized linear (seedling establishment, binomial error distribution) or linear mixed effect models testing the effect of species identity, soil conditioning, type of soil biota (soil filtrate vs whole-soil inoculum), conditioning of soil biota (from unconditioned vs self-conditioned soil), and their interactions on seedling establishment, plant biomass and root–shoot ratio

		Seedling establishment		Biomass		Root-shoot ratio	
	df	F	*p*	F	*p*	F	*p*
Species	1, 11	**52.21**	** < 0.001**	0.65	0.433	**29.70**	** < 0.001**
Soil conditioning [soil]	1, 89	0.04	0.843	**21.10**	** < 0.001**	**4.07**	**0.047**
Type of soil biota [biota type]	1, 88	0.47	0.492	**30.99**	** < 0.001**	**18.19**	** < 0.001**
Conditioning of soil biota [biota cond.]	1, 82	*3.85*	*0.050*	**4.13**	**0.045**	0.60	0.439
Species × soil	1, 89	*3.72*	*0.054*	0.29	0.594	**7.03**	**0.009**
Species × biota type	1, 88	0.09	0.767	0.53	0.469	0.16	0.692
Soil × biota type	1, 85	**9.05**	**0.003**	0.18	0.672	1.27	0.263
Species × biota cond	1, 82	1.19	0.276	**5.60**	**0.020**	**4.23**	**0.043**
Soil × biota cond	1, 89	0.01	0.936	**4.81**	**0.031**	1.28	0.261
Biota type × biota cond	1, 83	2.33	0.127	**11.44**	**0.001**	**16.28**	** < 0.001**
Species × soil × biota type	1, 85	*3.68*	*0.055*	0.94	0.334	1.77	0.187
Species × soil × biota cond	1, 88	2.00	0.157	2.50	0.117	0.15	0.700
Species × biota type × biota cond	1, 83	2.68	0.102	0.19	0.665	2.35	0.129
Soil × biota type × biota cond	1, 84	0.08	0.778	0.04	0.841	1.76	0.188
Species × soil × biota type × biota cond	1, 84	1.44	0.230	**4.15**	**0.044**	**4.65**	**0.034**

Significant results (*p* ≤ 0.05) are in bold, marginally significant (*p* ≤ 0.1) in italic. Df represent Satterthwaite approximation for degrees of freedom. The analyses are based on a subset of data excluding the sterilized and the whole soil treatments. Results of the analyses of a full dataset are shown in Table S3

**Fig. 3:**
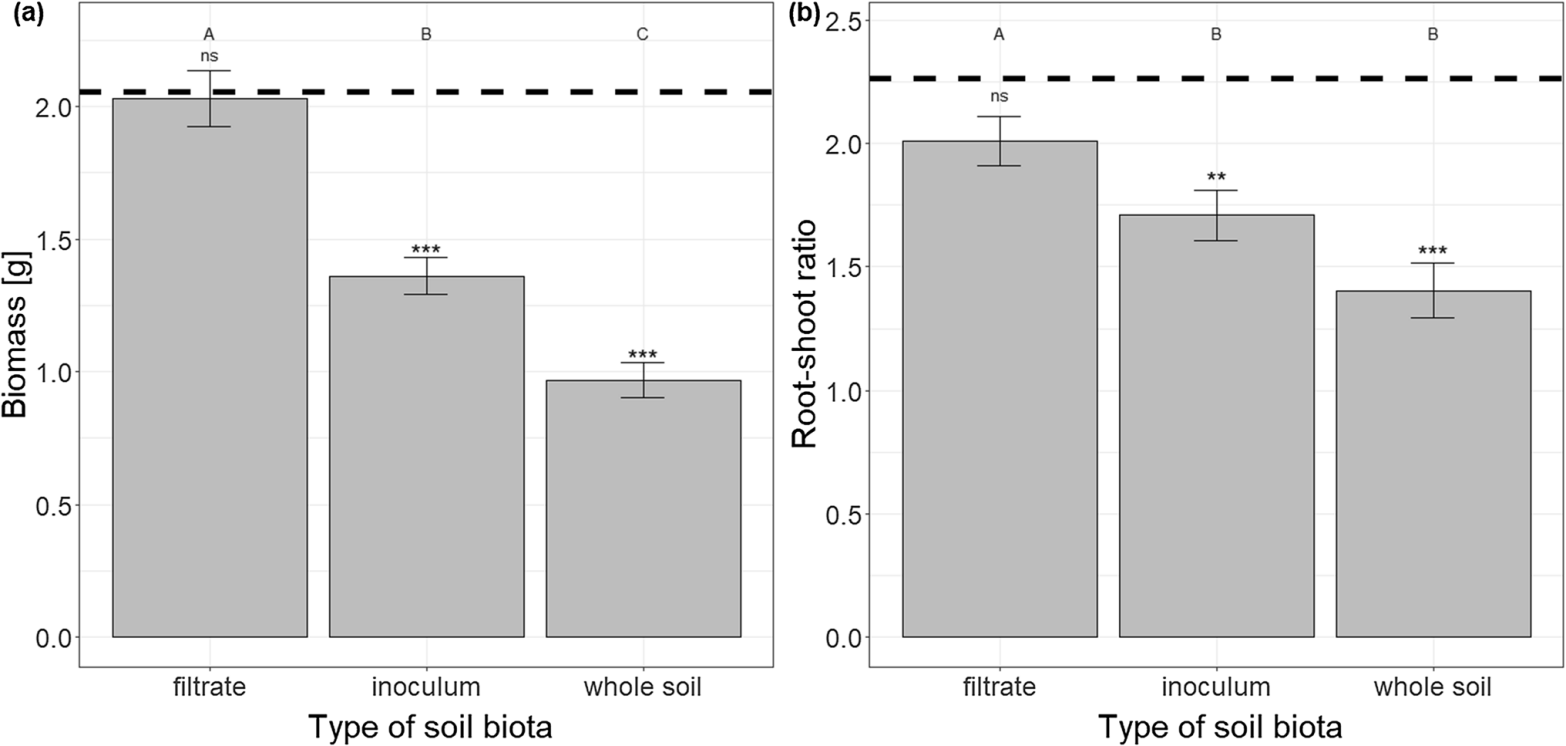
Effect of type of soil biota on **a** plant biomass and **b** root-shoot ratio across the two species. Bars and error lines represent mean ± SE. Bars that share the same letter do not significantly (*p* > 0.05) differ from each other after Tukey post-hoc tests. Dashed line represents mean value in the sterilized treatment, signs above the bars indicate whether the values differ from the sterilized treatment (***p < 0.001, **p < 0.01, ns *p* > 0.05)

Soil conditioning (i.e. conditioning of the background soil) negatively affected plant biomass, with no differences between the two species (Table 2, Fig. S4). Type of soil biota (i.e. soil filtrate, whole-soil inoculum, or whole soil) had a significant effect on plant biomass and root-shoot ratio (Table 2). In both cases, the values were the highest in soil filtrate treatments, lower in whole-soil inoculum and the lowest in whole soil (Fig. 3). The values were decreasing roughly by 25% per treatment for biomass and by 15% for root-shoot ratio. The effects did not differ between the two species (Table 2).

**Fig. 4:**
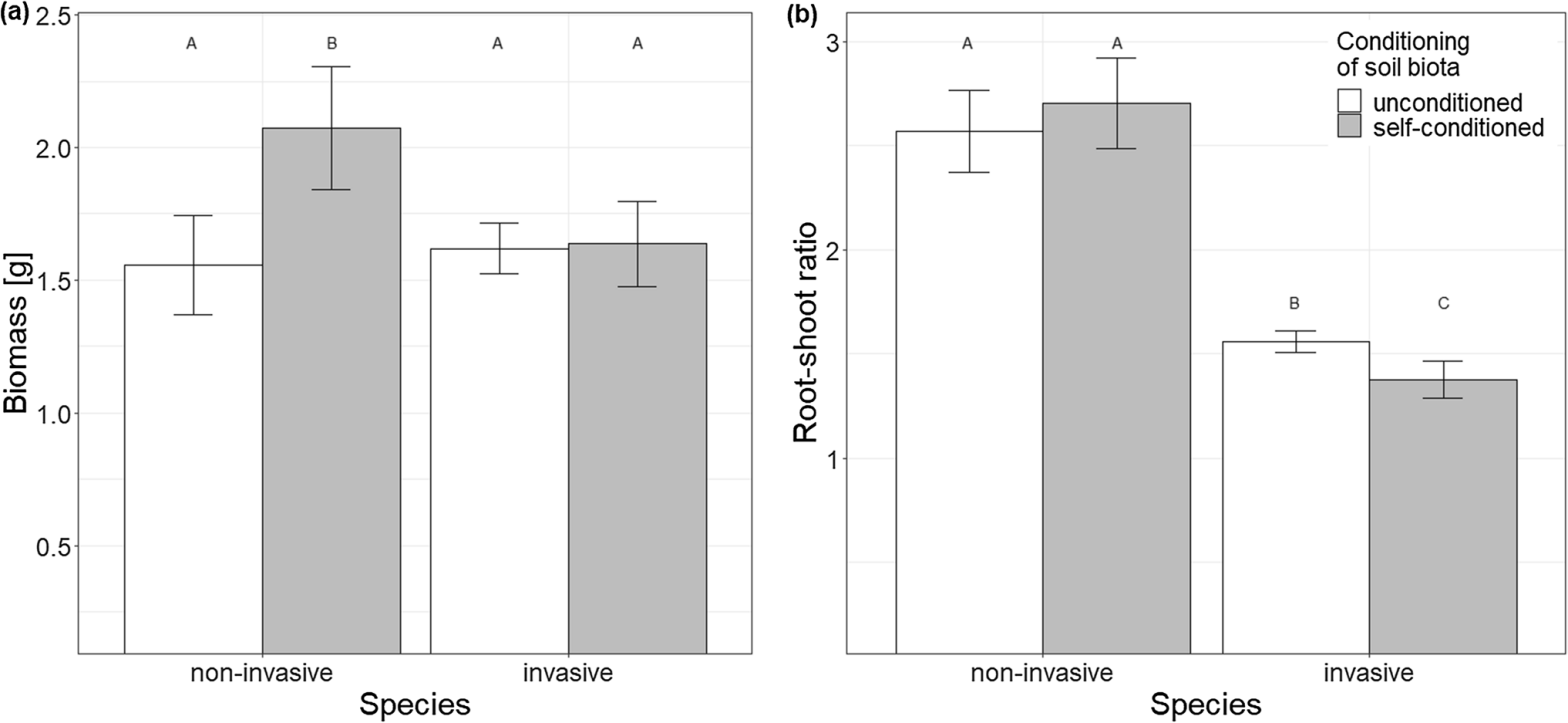
Effect of conditioning of soil biota on **a** biomass and **b** root-shoot ratio of the two study species. Bars and error lines represent mean ± SE. Bars that share the same letter do not significantly (*p* > 0.05) differ from each other after Tukey post-hoc tests

Effect of conditioning of soil biota, i.e. whether the biota (filtrate or inoculum) originated from unconditioned or self-conditioned soil, on biomass and root-shoot ratio differed between the two species (Table 2). When considering both filtrate and inoculum together, the non-invasive species had nearly by 30% higher biomass when grown with self-conditioned biota compared to biota from unconditioned soil, while the invasive species performed similarly with both self-conditioned and unconditioned biota (Fig. 4a). The root-shoot ratio of the non-invasive species did not differ when grown with self-conditioned and unconditioned biota, while the invasive species slightly, but significantly decreased its root-shoot ratio when grown with self-conditioned biota.

The interaction of species, type of soil biota and conditioning of soil biota was not significant for any of the measures of plant performance (Table 2). The interaction of species and treatment (comprising type and conditioning of soil biota) was significant for seedling establishment in the analysis using all treatments (Table S3). For the invasive species, seedling establishment in presence of unconditioned soil filtrate was approximately 10% higher (corresponding roughly to one extra seedling emerged) than in self-conditioned filtrate as well as the sterilized soil, but no differences in seedling establishment among the treatments were found for the non-invasive species (Fig. S5).

**Fig. 5:**
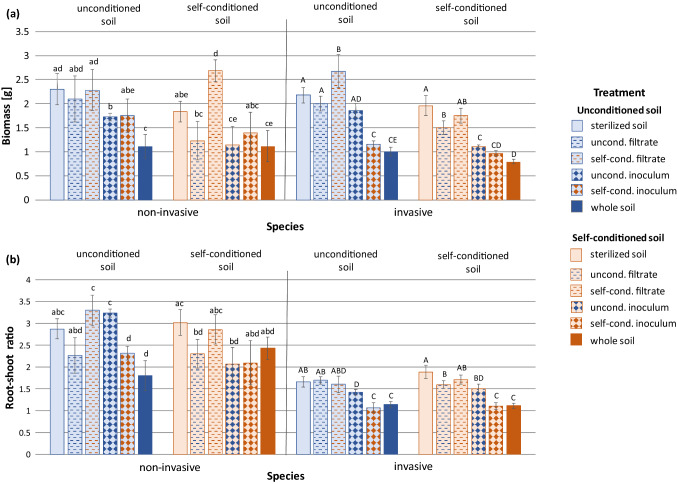
Effect of soil conditioning and treatment (type and conditioning of soil biota) on **a** biomass and **b** root–shoot ratio for individual species. Bars and error lines represent mean ± SE. Bars of one species that share the same letter do not significantly (*p* > 0.05) differ from each other after Tukey post-hoc tests

The interaction of species, soil conditioning, type of soil biota, and conditioning of soil biota was significant for biomass and root-shoot ratio (Table 2). In presence of self-conditioned soil filtrate, the non-invasive species had higher biomass than in the unconditioned soil filtrate in self-conditioned but not in unconditioned soil. The invasive species had higher biomass with self-conditioned filtrate than with unconditioned filtrate in unconditioned but not in self-conditioned soil (Fig. 5a). Root-shoot ratio was lower in plants grown with self-conditioned soil inoculum than with unconditioned soil inoculum in both unconditioned and self-conditioned soil for the invasive species, but only in unconditioned soil for the non-invasive species (Fig. 5b).

### Determinants of plant performance

**Fig. 6:**
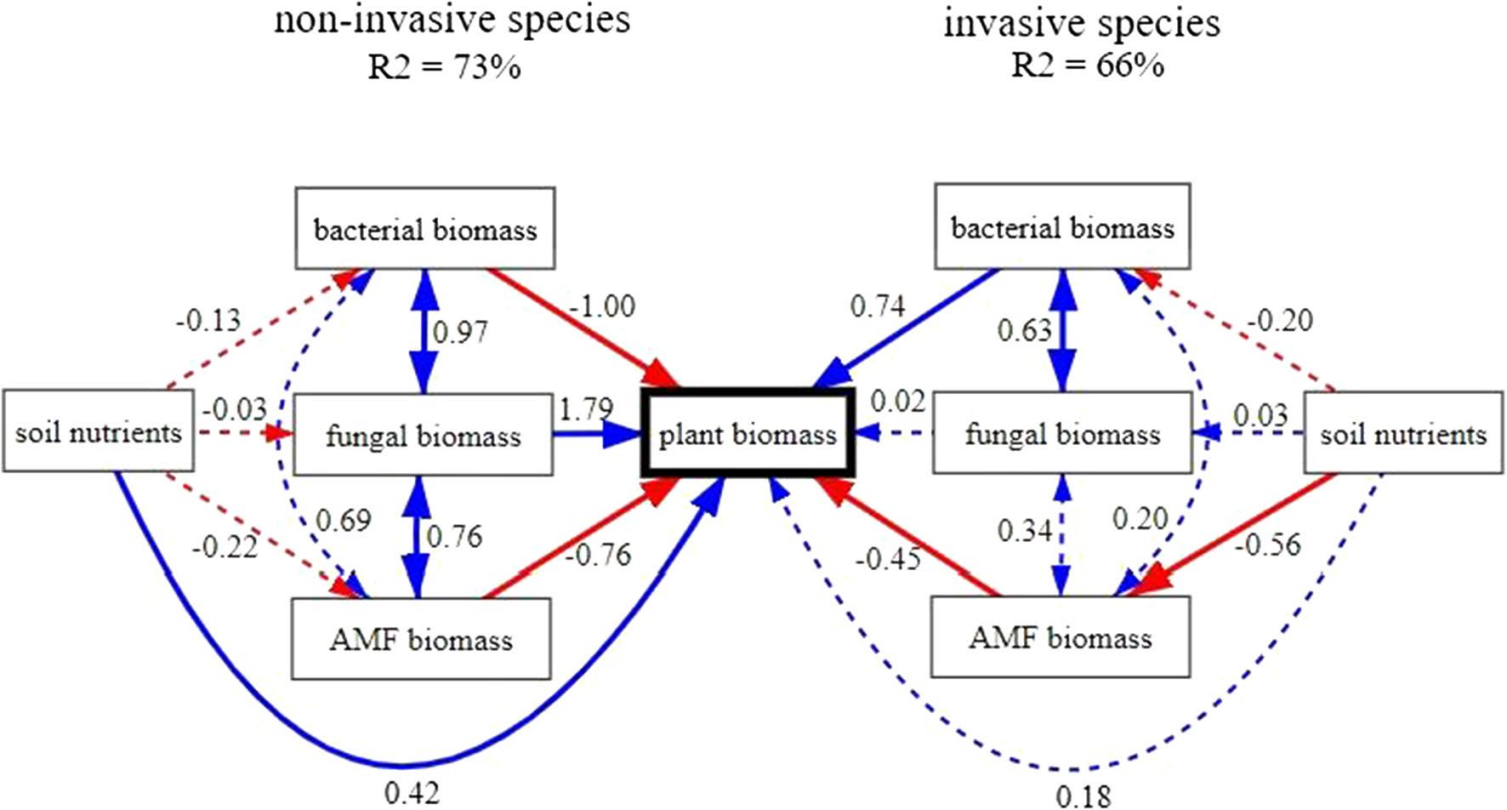
Structural equation models (path analysis) of soil characteristics influencing biomass of the non-invasive (panels on the left-hand side) and the invasive species (panels on the right-hand side). Red arrows indicate negative relationships, blue arrows positive relationships. Solid arrows indicate significant relationships (*P* < 0.05), dashed arrows non-significant relationships (*P* > 0.05). Standardized path coefficients are shown

Structural equation models (Fig. 6) showed that the determinants of plant performance in the whole soil treatment differ between the invasive and the non-invasive species. The non-invasive species responded negatively to bacterial biomass and positively to fungal biomass and soil nutrients levels, while the invasive species responded positively to bacterial biomass and was not significantly affected by fungal biomass or soil nutrients. While both species responded negatively to AMF biomass, only the invasive species significantly increased the AMF biomass as it depleted soil nutrients (Fig. 6).

## Discussion

In the present study, we compared plant–soil interactions in the native range of two congeneric plant species native to Europe that differ in their invasive status outside their native range –⁠ invasive *Cirsium vulgare* and non-invasive *C. oleraceum*. We showed that compared to its non-invasive congener, the invasive *C. vulgare* more rapidly depleted nutrients from the soil, was less influenced by availability of soil nutrients, and responded less positively / more negatively in terms of biomass and root-shoot ratio to presence of self-conditioned soil biota compared to unconditioned biota. The invasive species also had significantly higher seedling establishment, which increases its chances for successful invasion regardless of plant–soil interactions. Our results suggest that plant–soil interactions may play a role in the invasive potential of *C. vulgare* and highlight that experimental PSF studies in the native range of species can improve understanding of processes that regulate invasive plant populations.

According to our expectations, soils conditioned by the invasive species had lower levels of soil nutrients, particularly of available P, than soils conditioned by the non-invasive species (Fig. 2). This is in line with previous research showing that invasive species often exploit soil nutrients more efficiently than non-invasive species (Dassonville et al. 2008; Funk and Vitousek 2007; Sardans et al. 2017), allowing them to gain competitive advantage over other species. Importantly, the observed nutrient depletion in soils conditioned by the invasive species did not lead to a decrease in performance of the species (Fig. 5a, non-significant difference between performance in self-conditioned sterilized soil and unconditioned sterilized soil). In line with that, the structural equation models showed a lower sensitivity of the invasive species to soil nutrients than the non-invasive species (Fig. 6). This means that the invasive species copes better with altered nutrient levels, indicating its higher plasticity in response to nutrients, a common feature of successful invaders (Burns 2004; Daehler 2003; Funk 2008).

Biomass of both plant species decreased with increasing richness of soil biota (i.e. biomass was lower in presence of full soil biota from whole-soil inoculum than in presence of partial biota from soil filtrate Fig. 3a), showing that the negative effects of soil biota prevail over the positive effects, as has been shown in previous research (van de Voorde et al. 2012; Wang et al. 2019b). The invasive species responded more negatively to self-conditioned soil inoculum compared to the unconditioned inoculum than the non-invasive species (Fig. 5). This provides more opportunities for the invasive species to benefit from enemy release when introduced to the secondary range. These results are in line with the finding of Zuppinger-Dingley et al. (2011) that potentially invasive species are in their native range held in check by more negative response to self-conditioned soil biota compared to native species that do not become invasive elsewhere. Interestingly, both species in our study benefited from growth with self-conditioned soil filtrate compared to the unconditioned filtrate (Fig. 5a), and this was more pronounced for the non-invasive species. This result also helps with explaining the differences in success of the two species when introduced into a secondary range. When moving to the secondary range, the plants are supposed to leave their specialized soil biota behind and be mainly affected by the local, less specialized biota. The invasive *C. vulgare* seem to profit less from the specialized biota and thus has higher chance to profit from the non-specialized one.

Root-shoot ratio of both species decreased with increasing richness of soil biota (i.e. root-shoot ratio was lower in presence of full soil biota from whole-soil inoculum than in presence of partial biota from soil filtrate, Fig. 3b). In addition, the invasive species decreased its root-shoot ratio in presence of self-conditioned inoculum compared to unconditioned inoculum (Fig. 5b), pointing to its greater sensitivity to self-conditioned biota and possibly greater plasticity in biomass allocation compared to the non-invasive species. Since the size of root system determines the intensity of interactions between plants and soil biota (Aldorfova and Munzbergova 2019; Bergmann et al. 2016; Cortois et al. 2016), reducing allocation into root biomass in presence of detrimental soil biota may serve as a protective mechanism for the plants, minimizing the negative effects of soil biota on plant growth. On the other hand, reduced allocation to roots may have negative effect on plant growth in the long term via reduced ability to absorb nutrients, in case nutrients are limiting, and more research is thus needed to understand the relationship between allocation to roots and plant–soil interactions.

Seedling establishment was overall higher for the invasive species and did not differ between whole-soil inoculum treatment and sterilized soil for either species. Presence of unconditioned soil filtrate, however, increased seedling establishment of the invasive species (Fig. S5) compared to sterilized soil. This suggest that unconditioned biota from the filtrate, i.e. biota that is not effectively specialized to the species and is thus more likely to be encountered by the species in the secondary range, benefit plant performance of the invasive species in early stages of their life, but their positive effect is counterbalanced by soil pathogens when grown with self-conditioned biota from the native range. This, combined with the high overall seedling establishment, may be another factor contributing to the invasiveness of *C. vulgare*. However, our seedling establishment data were based only on 10 seeds sown per pot, so the results are not robust and should be interpreted with this limitation in mind.

While we cannot say exactly which groups of soil biota drive the changes in plant performance under individual treatments (see Methodological constrains below), some patterns were indicated by the structural equation models (Fig. 6). The models showed that bacterial biomass had an overall negative effect on the biomass of the non-invasive species, but positive on the invasive species, suggesting that the invasive species was less affected by bacterial pathogens and benefited more from bacterial mutualists. There is a large chance that the species benefits from presence of bacterial mutualists in the secondary range as well since most mutualists are quite generalist (Bronstein 2003) and invasive plants can often form mutualisms as effective or even more effective in the new ranges than in the old range (Parker and Gilbert 2007; Richardson et al. 2000).

The structural equation models (Fig. 6) further showed that AMF had a net negative effect on both plant species. Most studies find net positive effects of AMF on plants, however, several other studies have reported negative effects of AMF on plants (Janos 2007; Johnson et al. 1997), particularly in nutrient-rich soils such as the soil used in our experiment. For the invasive species, biomass of AMF was negatively correlated with level of soil nutrients (Fig. 6). Since the invasive species depleted nutrients from soil more efficiently than the non-invasive species, it also accumulated more AMF (Fig. 2), which had net negative effect on its further growth. Even though AMF have relatively low host specificity, host preference in natural ecosystems has been identified (Sanders 2003). Because AMF composition differs between world regions (Sturmer et al. 2018), chances are that the invasive species leaves behind some of the AMF when moving to the secondary range, which would further contribute to its invasion success. However, since we do not have data on AMF species composition or on the effect of AMF on the species in its secondary range, this explanation remains purely hypothetical.

### Methodological constrains

Using our experimental design allowed us to assess changes in soil chemistry and soil biota following soil conditioning by the two species as well as to evaluate the effects of conditioning the soil or its fractions on subsequent plant growth. However, our approach had its limitations. For example, we cannot say whether the differences in plant performance in unconditioned and self-conditioned soil were due to the differences in composition of the self-conditioned biota (i.e. more specific enemies) or just their abundance since both are likely to differ. We quantified the effects of overall bacterial, fungal and AMF biomass on plant growth in the conditioned soils and we compared plant performance in multiple treatments including sterilized soil and soil with unconditioned and self-conditioned filtrate or inoculum. However, we do not know which groups of soil biota contributed the most to the differences in performance under individual treatments, since we did not study composition of soil biota in soil filtrate and soil inoculum separately.

A possible limitation of our approach is also that we did not inoculate the soil with soil collected at natural localities of the study species as usually done in PSF studies. Our soil, therefore, included non-specific soil biota in the beginning of the experiment which was transitioned into more species-specific communities during the conditioning phase. However, even these more specific communities were still only a subset of biota originally found in the mixture of topsoil and sand and may thus not reflect soil microbial community of either species in natural conditions. On the other hand, the approach simulates the introduction of the species into a new environment, which is what both our study species commonly experience when colonizing disturbed habitats or invading new sites, and use of the soil is therefore justified in context of our study.

## Conclusions

By comparing plant–soil interactions of globally invasive *Cirsium vulgare* and its non-invading congener *C. oleraceum*, we showed that plant–soil interactions in their native range may help to explain the differences in the invasive success of the species. This invasive species is able to reduce nutrients to lower levels but maintain its high performance regardless of soil nutrient levels. While soil bacteria in general have more positive effect on the invasive species, the invasive species benefits less from growth with self-conditioned biota transferred by soil filtrate compared to unconditioned biota. On the other hand, it is relatively more harmed by self-conditioned biota transferred by soil inoculum. Since the self-conditioned biota is likely more specialized and, therefore, less likely to be present in the secondary range than the unconditioned biota, our results suggest that the invasive species may benefit more from pathogen release and at the same time suffer less from loss of specialized mutualists when transferred to the secondary range than the non-invasive species. Further studies considering more complex interactions — such as experiments including heterospecific plant–soil interactions (Bever et al. 1997) along with conspecific plant–soil interactions studied here or experiments combining plant–soil interaction treatments with intra- and/or inter-specific competition treatments (Dostalek et al. 2022; Lekberg et al. 2018; Shannon et al. 2012) –— are needed to account for context dependency of PSF studies and to provide a deeper insight into environmental constraints limiting performance of both species in their native and secondary ranges.

## Supplementary Information

Below is the link to the electronic supplementary material.

Supplementary file1 (DOCX 292 KB)

## Data Availability

The datasets used and analyzed during the current study are available from the corresponding author on reasonable request.

## References

[CR1] Adelman MJ, Morton JB (1986). Infectivity of vesicular arbuscular mycorrhizal fungi - influence of host soil diluent combinations on mpn estimates and percentage colonization. Soil Biol Biochem.

[CR2] Aldorfova A, Munzbergova Z (2019). Conditions of plant cultivation affect the differences in intraspecific plant-soil feedback between invasive and native dominants. Flora.

[CR3] Aldorfova A, Knobova P, Munzbergova Z (2020). Plant-soil feedback contributes to predicting plant invasiveness of 68 alien plant species differing in invasive status. Oikos.

[CR4] Aldorfova A, Dostalek T, Munzbergova Z (2022). Effects of soil conditioning, root and shoot litter addition interact to determine the intensity of plant-soil feedback. Oikos.

[CR5] Ammerman J (2001) Determination of Nitrate/Nitrite in 0,5 M K_2_SO_4_ soil extracts by Flow Injection analysis. QuikChem Method 12-107-04-1-H.

[CR6] Bergmann J, Verbruggen E, Heinze J, Xiang D, Chen BD, Joshi J, Rillig MC (2016). The interplay between soil structure, roots, and microbiota as a determinant of plant-soil feedback. Ecol Evol.

[CR7] Bever JD, Westover KM, Antonovics J (1997). Incorporating the soil community into plant population dynamics: the utility of the feedback approach. J Ecol.

[CR8] Bligh EG, Dyer WJ (1959). A rapid method of total lipid extraction and purification. Can J Biochem Physiol.

[CR10] Brinkman EP, Van der Putten WH, Bakker EJ, Verhoeven KJF (2010). Plant-soil feedback: experimental approaches, statistical analyses and ecological interpretations. J Ecol.

[CR11] Bronstein JL (2003) The scope for exploitation within mutualistic interactions. Genetic and Cultural Evolution of Cooperation: 185–202

[CR12] Burns JH (2004). A comparison of invasive and non-invasive dayflowers (Commelinaceae) across experimental nutrient and water gradients. Divers Distrib.

[CR13] Callaway RM, Bedmar EJ, Reinhart KO, Silvan CG, Klironomos J (2011). Effects of soil biota from different ranges on Robinia invasion: acquiring mutualists and escaping pathogens. Ecology.

[CR14] Chiuffo MC, Macdougall AS, Hierro JL (2015). Native and non-native ruderals experience similar plant-soil feedbacks and neighbor effects in a system where they coexist. Oecologia.

[CR15] Cortois R, Schroder-Georgi T, Weigelt A, van der Putten WH, De Deyn GB (2016). Plant-soil feedbacks: role of plant functional group and plant traits. J Ecol.

[CR16] Daehler CC (2003). Performance comparisons of co-occurring native and alien invasive plants: Implications for conservation and restoration. Annu Rev Ecol Evol Syst.

[CR17] Dassonville N, Vanderhoeven S, Vanparys V, Hayez M, Gruber W, Meerts P (2008). Impacts of alien invasive plants on soil nutrients are correlated with initial site conditions in NW Europe. Oecologia.

[CR18] Dawson W, Schrama M (2016). Identifying the role of soil microbes in plant invasions. J Ecol.

[CR19] Dědina J (1987) Selected methods of analytic atom spectrochemistry. Československá spektroskopická společnost

[CR20] Dostalek T, Knappova J, Munzbergova Z (2022). The role of plant-soil feedback in long-term species coexistence cannot be predicted from its effects on plant performance. Ann Bot.

[CR21] Dudenhoffer JH, Ebeling A, Klein AM, Wagg C (2018). Beyond biomass: Soil feedbacks are transient over plant life stages and alter fitness. J Ecol.

[CR22] Egan L (2001) Determination of Amonia by Flow Injection Analysis Colorimetry. QuikChem Metod 10-107-06-5-E.

[CR23] Ehrenberger F, Gorbach S (1973) Methoden der organischen Elementar- und Spurenanalyse. Verlag Chemie Weinheim

[CR24] Enders M, Havemann F, Ruland F, Bernard-Verdier M, Catford JA, Gomez-Aparicio L, Haider S, Heger T, Kueffer C, Kuhn I, Meyerson LA, Musseau C, Novoa A, Ricciardi A, Sagouis A, Schittko C, Strayer DL, Vila M, Essl F, Hulme PE, van Kleunen M, Kumschick S, Lockwood JL, Mabey AL, McGeoch MA, Palma E, Pysek P, Saul WC, Yannelli FA, Jeschke JM (2020). A conceptual map of invasion biology: Integrating hypotheses into a consensus network. Glob Ecol Biogeogr.

[CR25] Engelkes T, Morrien E, Verhoeven KJF, Bezemer TM, Biere A, Harvey JA, McIntyre LM, Tamis WLM, van der Putten WH (2008). Successful range-expanding plants experience less above-ground and below-ground enemy impact. Nature.

[CR26] Feldmann F, Idczak E (1992). Inoculum production of vesicular-arbuscular mycorrhizal fungi for use in tropical nurseries. Methods Microbiol.

[CR27] Florianova A, Munzbergova Z (2018). The intensity of intraspecific plant-soil feedbacks in alien Impatiens species depends on the environment. Perspect Plant Ecol Evolut System.

[CR28] Funk JL (2008). Differences in plasticity between invasive and native plants from a low resource environment. J Ecol.

[CR29] Funk JL, Vitousek PM (2007). Resource-use efficiency and plant invasion in low-resource systems. Nature.

[CR30] Gallery RE, Dalling JW, Arnold AE (2007). Diversity, host affinity, and distribution of seed-infecting fungi: A case study with Cecropia. Ecology.

[CR31] Garcia-Sanchez M, Cajthaml T, Filipova A, Tlustos P, Szakova J, Garcia-Romera I (2019). Implications of mycoremediated dry olive residue application and arbuscular mycorrhizal fungi inoculation on the microbial community composition and functionality in a metal-polluted soil. J Environ Manag.

[CR32] Gioria M, Osborne BA (2014). Resource competition in plant invasions: emerging patterns and research needs. Front Plant Sci.

[CR33] Giovannetti M, Mosse B (1980). An evaluation of techniques for measuring vesicular arbuscular mycorrhizal infection in roots. New Phytol.

[CR34] Hannula SE, Heinen R, Huberty M, Steinauer K, De Long JR, Jongen R, Bezemer TM (2021). Persistence of plant-mediated microbial soil legacy effects in soil and inside roots. Nat Commun.

[CR35] Hendriks M, Ravenek JM, Smit-Tiekstra AE, van der Paauw JW, de Caluwe H, van der Putten WH, de Kroon H, Mommer L (2015). Spatial heterogeneity of plant-soil feedback affects root interactions and interspecific competition. New Phytol.

[CR36] Hothorn T, Bretz F, Westfall P (2008). Simultaneous inference in general parametric models. Biom J.

[CR37] Janos DP (2007). Plant responsiveness to mycorrhizas differs from dependence upon mycorrhizas. Mycorrhiza.

[CR38] Johnson NC, Graham JH, Smith FA (1997). Functioning of mycorrhizal associations along the mutualism-parasitism continuum. New Phytol.

[CR39] Julien MH, Griffiths MW (1998) Biological control of weeds. A world catalogue of agents and their target weeds. CABI Publishing, Wallingford, UK

[CR40] Kardol P, Cornips NJ, van Kempen MML, Bakx-Schotman JMT, van der Putten WH (2007). Microbe-mediated plant-soil feedback causes historical contingency effects in plant community assembly. Ecol Monogr.

[CR41] Kardol P, De Deyn GB, Laliberte E, Mariotte P, Hawkes CV (2013). Biotic plant-soil feedbacks across temporal scales. J Ecol.

[CR42] Keane RM, Crawley MJ (2002). Exotic plant invasions and the enemy release hypothesis. Trends Ecol Evol.

[CR43] Klironomos JN (2002). Feedback with soil biota contributes to plant rarity and invasiveness in communities. Nature.

[CR44] Kulmatiski A, Beard KH, Stevens JR, Cobbold SM (2008). Plant-soil feedbacks: a meta-analytical review. Ecol Lett.

[CR45] Kulmatiski A, Heavilin J, Beard KH (2011). Testing predictions of a three-species plant-soil feedback model. J Ecol.

[CR46] Kulmatiski A, Kardol P (2008) Getting plant–soil feedbacks out of the greenhouse: experimental and conceptual approaches In: Uea Lüttge (Ed) Progress in Botany. Springer-Verlag, Berlin Heidelberg.

[CR47] Kuznetsova A, Brockhoff PB, Christensen RHB (2017). lmerTest package: tests in linear mixed effects models. J Stat Softw.

[CR48] Lekberg Y, Bever JD, Bunn RA, Callaway RM, Hart MM, Kivlin SN, Klironomos J, Larkin BG, Maron JL, Reinhart KO, Remke M, van der Putten WH (2018). Relative importance of competition and plant-soil feedback, their synergy, context dependency and implications for coexistence. Ecol Lett.

[CR49] Lepinay C, Vondrakova Z, Dostalek T, Munzbergova Z (2018). Duration of the conditioning phase affects the results of plant-soil feedback experiments via soil chemical properties. Oecologia.

[CR50] Mathakutha R, Steyn C, le Roux PC, Blom IJ, Chown SL, Daru BH, Ripley BS, Louw A, Greve M (2019). Invasive species differ in key functional traits from native and non-invasive alien plant species. J Veg Sci.

[CR51] McGinn KJ, van der Putten WH, Hulme PE, Shelby N, Weser C, Duncan RP (2018). The influence of residence time and geographic extent on the strength of plant-soil feedbacks for naturalised Trifolium. J Ecol.

[CR52] Meijer SS, Holmgren M, Van der Putten WH (2011). Effects of plant-soil feedback on tree seedling growth under arid conditions. J Plant Ecol.

[CR53] Montesinos D, Callaway RM (2020). Soil origin corresponds with variation in growth of an invasiveCentaurea, but not of non-invasive congeners. Ecology.

[CR54] Moore PD, Chapman SB (1986). Methods in plant ecology.

[CR55] Moorman T, Reeves FB (1979). Role of endomycorrhizae in revegetation practices in the semi-arid West.2. bioassay to determine the effect of land disturbance on endomycorrhizal populations. Am J Bot.

[CR56] Olsen SR, Sommers LE (1982) Phosphorus. In: Page AL, Miller RH, Keeney DR (Eds) Methods of soil analysis, part 2, Chemical and microbiological properties. American Society of Agronomy, Madison

[CR57] Olsson PA, Larsson L, Bago B, Wallander H, van Aarle IM (2003). Ergosterol and fatty acids for biomass estimation of mycorrhizal fungi. New Phytol.

[CR58] Oono R, Black D, Slessarev E, Sickler B, Strom A, Apigo A (2020). Species diversity of fungal endophytes across a stress gradient for plants. New Phytol.

[CR59] Parker IM, Gilbert GS (2007). When there is no escape: The effects of natural enemies on native, invasive, and noninvasive plants. Ecology.

[CR60] Peacher MD, Meiners SJ (2020). Inoculum handling alters the strength and direction of plant-microbe interactions. Ecology.

[CR61] Perkins LB, Nowak RS (2013). Native and non-native grasses generate common types of plant-soil feedbacks by altering soil nutrients and microbial communities. Oikos.

[CR62] R Core Team A (2019) R: A Language and Environment for Statistical Computing. R Foundation for Statistical Computing, Vienna, Austria

[CR63] Reinhart KO, Callaway RM (2004). Soil biota facilitate exotic Acer invasions in Europe and North America. Ecol Appl.

[CR64] Reinhart KO, Packer A, Van der Putten WH, Clay K (2003). Plant-soil biota interactions and spatial distribution of black cherry in its native and invasive ranges. Ecol Lett.

[CR65] Richardson DM, Allsopp N, D'Antonio CM, Milton SJ, Rejmanek M (2000). Plant invasions - the role of mutualisms. Biol Rev.

[CR66] Rinella MJ, Reinhart KO (2019). Toward more robust plant-soil feedback research: reply. Ecology.

[CR67] Rosseel Y (2012). lavaan: An R package for structural equation modeling. J Stat Softw.

[CR68] Sampedro I, Giubilei M, Cajthaml T, Federici E, Federici F, Petruccioli M, D'Annibale A (2009). Short-term impact of dry olive mill residue addition to soil on the resident microbiota. Biores Technol.

[CR69] Sanders IR (2003). Preference, specificity and cheating in the arbuscular mycorrhizal symbiosis. Trends Plant Sci.

[CR70] Sardans J, Bartrons M, Margalef O, Gargallo-Garriga A, Janssens IA, Ciais P, Obersteiner M, Sigurdsson BD, Chen HYH, Penuelas J (2017). Plant invasion is associated with higher plant-soil nutrient concentrations in nutrient-poor environments. Glob Change Biol.

[CR71] Semchenko M, Barry KE, de Vries FT, Mommer L, Moora M, Macia-Vicente JG (2022). Deciphering the role of specialist and generalist plant-microbial interactions as drivers of plant-soil feedback. New Phytol.

[CR72] Shannon S, Flory SL, Reynolds H (2012). Competitive context alters plant-soil feedback in an experimental woodland community. Oecologia.

[CR73] Sieg CH, Phillips BG, Moser LP (2003) Exotic invasive plants. In: Frederici P (Ed) Ecological Restoration of Southwestern Ponderosa Pine Forests. Island Press, Washington, DC.

[CR74] Snajdr J, Valaskova V, Merhautova V, Cajthaml T, Baldrian P (2008). Activity and spatial distribution of lignocellulose-degrading enzymes during forest soil colonization by saprotrophic basidiomycetes. Enzyme Microb Technol.

[CR75] Sturmer SL, Bever JD, Morton JB (2018). Biogeography of arbuscular mycorrhizal fungi (Glomeromycota) a phylogenetic perspective on species distribution patterns. Mycorrhiza.

[CR76] Suding KN, Harpole WS, Fukami T, Kulmatiski A, MacDougall AS, Stein C, van der Putten WH (2013). Consequences of plant-soil feedbacks in invasion. J Ecol.

[CR77] Tenhumberg B, Louda SM, Eckberg JO, Takahashi M (2008). Monte Carlo analysis of parameter uncertainty in matrix models for the weed Cirsium vulgare. J Appl Ecol.

[CR9] ter Braak CJ, Šmilauer P (2012) Canoco reference manual and user’s guide: software for ordination, version 5.0. Microcomputer Power, Ithaca

[CR78] van de Voorde TFJ, van der Putten WH, Bezemer TM (2011). Intra- and interspecific plant-soil interactions, soil legacies and priority effects during old-field succession. J Ecol.

[CR79] van de Voorde TFJ, van der Putten WH, Bezemer TM (2012). Soil inoculation method determines the strength of plant-soil interactions. Soil Biol Biochem.

[CR80] van der Putten WH, Klironomos JN, Wardle DA (2007). Microbial ecology of biological invasions. ISME J.

[CR81] van Grunsven RHA, van der Putten WH, Bezemer TM, Tamis WLM, Berendse F, Veenendaal EM (2007). Reduced plant-soil feedback of plant species expanding their range as compared to natives. J Ecol.

[CR82] van Grunsven RHA, van der Putten WH, Bezemer TM, Veenendaal EM (2010). Plant-soil feedback of native and range-expanding plant species is insensitive to temperature. Oecologia.

[CR83] Wagg C, Bender SF, Widmer F, van der Heijden MGA (2014). Soil biodiversity and soil community composition determine ecosystem multifunctionality. Proc Natl Acad Sci USA.

[CR84] Wagg C, Schlaeppi K, Banerjee S, Kuramae EE, van der Heijden MGA (2019). Fungal-bacterial diversity and microbiome complexity predict ecosystem functioning. Nat Commun.

[CR85] Wang P, Zhang XY, Kong CH (2013). The response of allelopathic rice growth and microbial feedback to barnyardgrass infestation in a paddy field experiment. Eur J Soil Biol.

[CR86] Wang MG, De Deyn GB, Bezemer TM (2019). Separating effects of soil microorganisms and nematodes on plant community dynamics. Plant Soil.

[CR87] Wang MG, Ruan WB, Kostenko O, Carvalho S, Hannula SE, Mulder PPJ, Bu FJ, van der Putten WH, Bezemer TM (2019). Removal of soil biota alters soil feedback effects on plant growth and defense chemistry. New Phytol.

[CR88] Wild J, Kaplan Z, Danihelka J, Petrik P, Chytry M, Novotny P, Rohn M, Sule V, Bruna J, Chobot K, Ekrt L, Holubova D, Knollova I, Kocian P, Stech M, Stepanek J, Zouhar V (2019). Plant distribution data for the Czech Republic integrated in the Pladias database. Preslia.

[CR89] Wilschut RA, van Kleunen M (2021). Drought alters plant-soil feedback effects on biomass allocation but not on plant performance. Plant Soil.

[CR90] Wilson JM, Trinick MJ (1983). Factors affecting the estimation of numbers of infective propagules of vesicular arbuscular mycorrhizal fungi by the most probable number method. Aust J Soil Res.

[CR91] Zuppinger-Dingley D, Schmid B, Chen Y, Brandl H, van der Heijden MGA, Joshi J (2011). In their native range, invasive plants are held in check by negative soil-feedbacks. Ecosphere.

