# Supplementary material for: Plant–soil interactions in the native range of two congeneric species with contrasting invasive success
Source: Oecologia. 2023 Feb 6;201(2):461–77. doi: 10.1007/s00442-023-05329-6 (PMC9945059; doi:10.1007/s00442-023-05329-6)
Supplement: Supplementary file 1 — Supplementary file1 (DOCX 292 KB) [file 442_2023_5329_MOESM1_ESM.docx]

**Plant-soil interactions in the native range of two congeneric species with contrasting invasive success**

Anna Florianová^1,2*^, Věra Hanzelková^1,2^, Lucie Drtinová^1^, Hana Pánková^2^, Tomáš Cajthaml^3,4^, Zuzana Münzbergová^1,2^

^1^Department of Botany, Faculty of Science, Charles University in Prague, Benátská 2, 128 01 Praha 2, Czech Republic

^2^Institute of Botany of the Czech Academy of Sciences, Zámek 1, 252 43 Průhonice, Czech Republic

^3^Institute of Microbiology of the Czech Academy of Sciences, Vídeňská 1083, 142 20 Praha 4, Czech Republic

^4^Institute for Environmental Studies, Faculty of Science, Charles University in Prague, Benátská 2, 128 01 Praha 2, Czech Republic

**Supplementary Information**

**Table S1** Abiotic characteristics of the soil prior to the conditioning phase. Values show the mean and standard deviation of six samples. The analyses were performed by the Analytical Laboratory of Institute of Botany, Czech Academy of Sciences, Průhonice

|  | mean ± sd |
| --- | --- |
| actual pH | 7.87 ± 0.03 |
| exchangeable pH | 7.62 ± 0.08 |
| total N [%] | 0.07 ± 0.02 |
| total C [%] | 0.91 ± 0.26 |
| total P [mg/kg] | 204.98 ± 35.04 |
| available P [mg/kg] | 35.99 ± 2.15 |
| available Ca [mg/kg] | 1691.77 ± 134.60 |
| available Mg [mg/kg] | 117.11 ± 11.47 |
| available K [mg/kg] | 272.91 ± 24.67 |

**Table S2** Correlation matrix of soil characteristics. C, N and total P refer to total content of the nutrients, available P, Ca, Mg, and K to content of available nutrients. Total microbes, bacteria (B), actinobacteria, Gram+ (Gram-positive bacteria, G+), Gram- (Gram-negative bacteria, G-), fungi (F), and AMF represent biomass of the individual groups of soil biota obtained by PLFA and NLFA analyses, and F/B, G+/G-, F/AMF their ratios as described in Material and Methods. Significant correlations (p < 0.05) are in bold, positive correlations are highlighted by blue and negative correlations by red color. Characteristics denoted with an asterisk were not used in the analyses due to their high correlation with other soil characteristics

**Table S3** Results of generalized linear (seedling establishment, binomial error distribution) or linear mixed effect models testing the effect of species identity, soil conditioning, treatment, and their interactions on seedling establishment, plant biomass and root-shoot ratio. Significant results (p ≤ 0.05) are in bold, marginally significant (p ≤ 0.1) in italic. Df represent Satterthwaite approximation for degrees of freedom. The analysis is based on a complete dataset including all 12 treatments

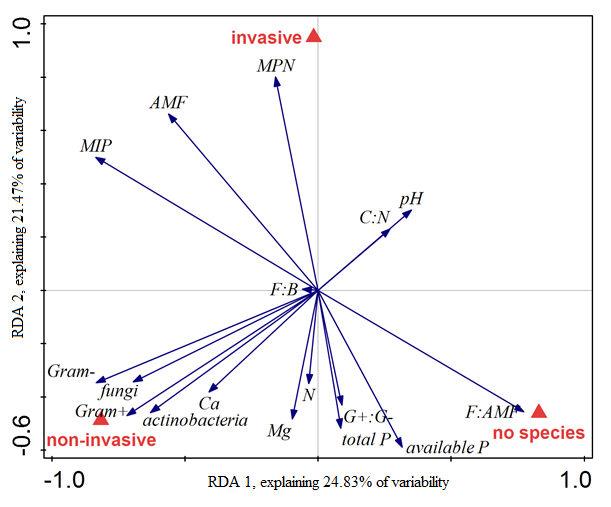


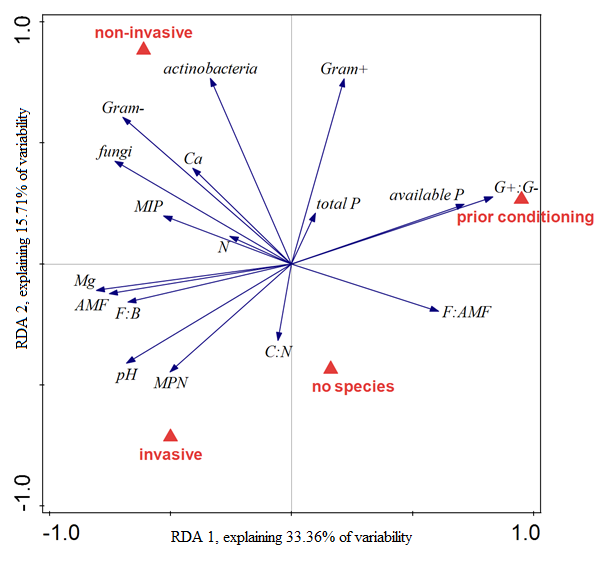


**Fig. S1** Differences in soil biotic and abiotic characteristics (a) in soils conditioned by the invasive and the non-invasive species and in unconditioned soil (no species), and (b) in soil prior to soil conditioning as well. Results displayed are an ordination plot RDA tested using a Monte-Carlo test with 499 permutations. Results are centered and standardized across soil characteristics. In (a) the first two axes explained 24.83% and 21.47% of variability in the data, respectively, Pseudo-F = 6.5, p = 0.002; in (b) 33.36% and 15.71% of variability in the data, Pseudo-F = 10.3, p = 0.002. Actinobacteria, Gram+, Gram-, fungi and AMF represent biomass of the groups obtained by PLFA/NLFA analyses. G+:G-, F:AMF, and F:B represent ratios of Gram positive (G+) and Gram negative (G-) bacteria, fungi (F), AMF and bacteria (B)


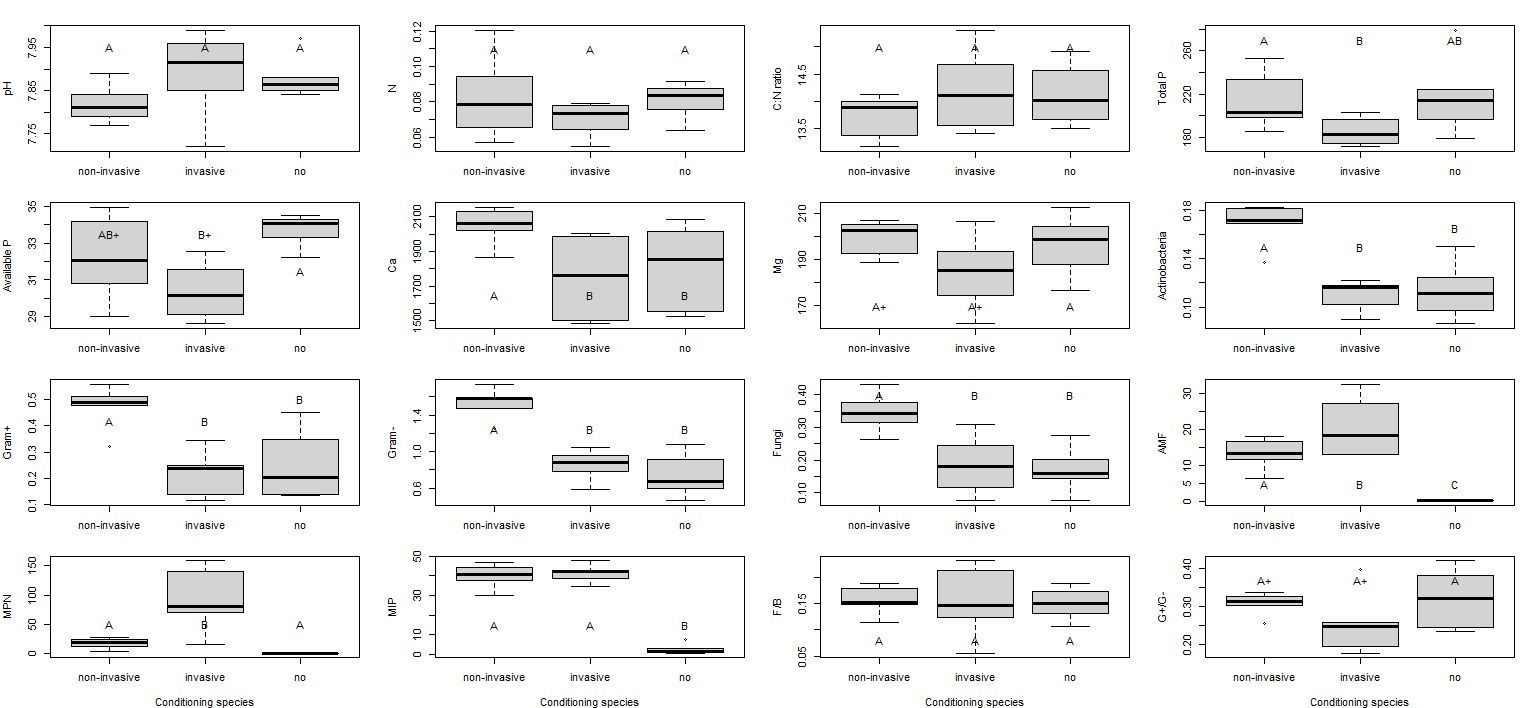


**Fig. S2** Differences of individual soil characteristics in soils conditioned by the invasive and the non-invasive species, and in unconditioned soil (no species). Boxes that share the same letter do not significantly (P > 0.05) differ from each other after Tukey post-hoc tests, differences between boxes marked with a + are marginally significant (0.05 < P < 0.1)


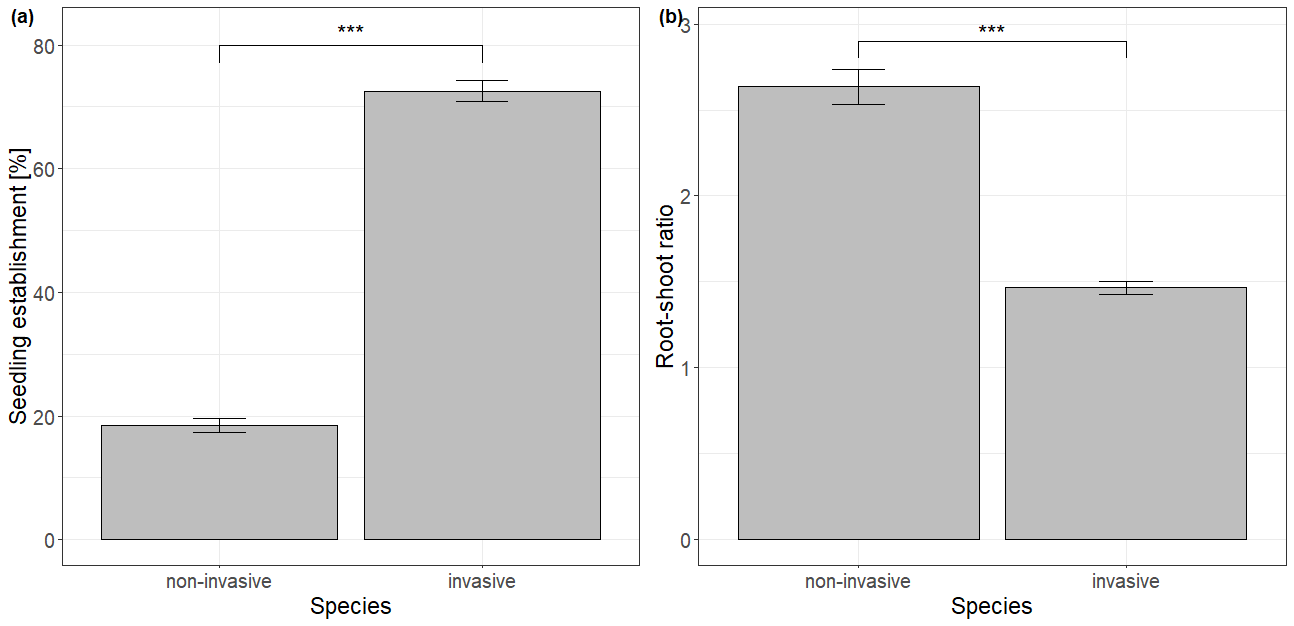


**Fig. S3** Differences in (a) seedling establishment and (b) root-shoot ratio between the two study species. Bars and error lines represent mean ± SE. Significant differences between bars are indicated by asterisks (*** p < 0.001)


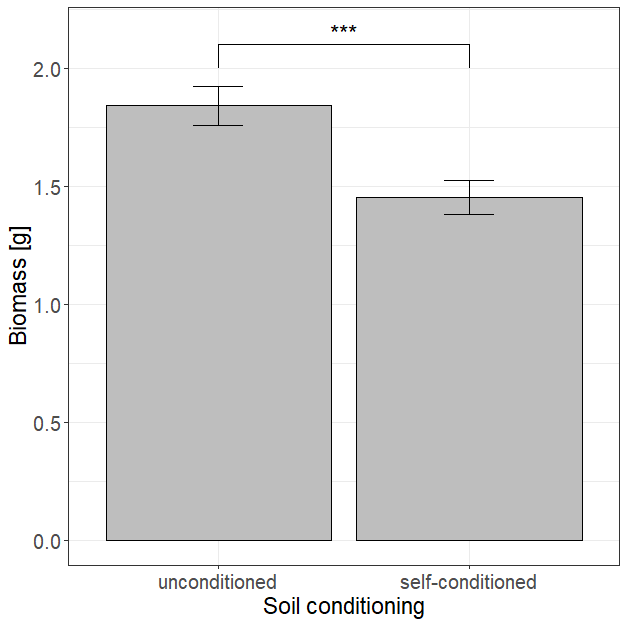


**Fig. S4** Effect of soil conditioning on plant biomass. Bars and error lines represent mean ± SE. Significant differences between bars are indicated by asterisks (*** p < 0.001)

**Fig. S5** Effect of treatment (type and conditioning of soil biota) on seedling establishment for individual species. Bars and error lines represent mean ± SE. Bars that share the same letter do not significantly (p > 0.05) differ from each other after Tukey post-hoc tests (tested separately for each species)
